# Thermoregulation as a non-unified system: A difficult to teach concept

**DOI:** 10.1080/23328940.2017.1281872

**Published:** 2017-01-19

**Authors:** Luca Imeri

**Affiliations:** Department of Health Sciences, University of Milan Medical School, San Paolo University Hospital Via A. di Rudinì, 8, 20142 Milan, Italyluca.imeri@unimi.it

## Thermoregulation with a thermostat

**Figure 1.**
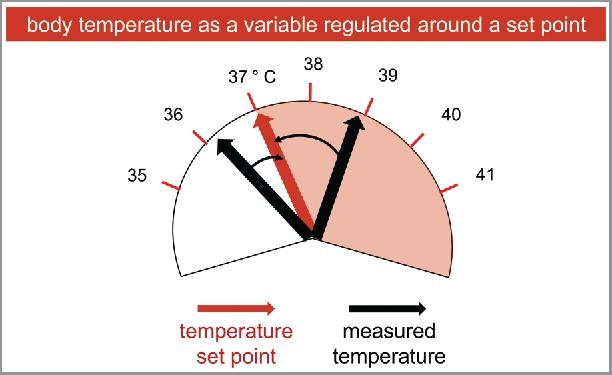
Thermoregulation in a unified system.

I am a physiologist, but not a thermophysiologist. My whole research has focused on something completely different (i.e. - just to give you an idea of how far is my field - the basic neurobiology of brain circuits involved in sleep regulation^1^). But as an associate professor of physiology at an Italian University (the University of Milan Medical School) I teach the “whole” physiology, from the neuron to the nephron (as sometime I joke with friends and colleagues). And somewhere between the neuron and the nephron there is thermophysiology, something that I used to think as (very) easy to teach. Even more, one of the best example of a homeostatic control circuit: you have a set point, somehow hardwired in a specific brain region (let's simplify a little bit and say the anterior hypothalamus), which functions as the control center (a central commander). You also have central (within the brain) and peripheral (outside the brain) sensors, which inform the control center of the actual temperature in these districts. This information is put together, i. e. integrated. If the measured temperature corresponds to the set point, nothing is done. On the other hand, if measured temperature does not correspond to the set point, the control center orchestrates the coordinated activation of the appropriate temperature-increasing or temperature-decreasing mechanisms to bring the measured temperature back to the set point value. To illustrate these concepts, I used to present the slide in Figure 1. What is difficult in this “story”? Nothing: it is a simple, clear, and, at least in my opinion, (very) powerful description.

## Thermoregulation without a thermostat

But, a few years ago, I organized, for the Ph.D. students in the Neuroscience program of our University, a one day-course on thermophysiology and invited, as Faculties, Prof. Romanovsky (the Editor in Chief of this journal) and Prof. Parmeggiani (from the University of Bologna). At that point, I had to brush up my thermophysiology a little bit. So I had the chance to find out that a few concepts had changed^3^ since the last time I had perused the subject. Somebody was proposing that body temperature is NOT “regulated by a unified system with a single controller,” but by “independent thermoeffector loops, each having its own afferent and efferent branches.”^3^ I started scratching my head. Moreover: “No computation of an integrated body temperature or its comparison with an obvious or hidden set point of a unified system is necessary. Coordination between thermoeffectors is achieved through their common controlled variable, body temperature.”^3^ I was immediately fascinated by (I could even say hooked to) the idea of a highly sophisticated and efficient regulatory system that would/could work without a “central commander,” but as the result of the interaction between different, but related forces. I will come back to the concept of a regulatory, but not unified system later (please see “From top-down to bottom-up models”).

While I kept scratching my head, I started thinking of something like a cane stuck into the ground, with ropes pulling the cane in opposite directions: the position of the cane stuck into the ground would depend on the balance between the forces pulling on it in different directions. The cane would probably be stuck, but somehow loose into the ground, as our temperature changes, but we are built (i. e. we evolved) to maintain (as much as possible) a given temperature.

**Figure 2.**
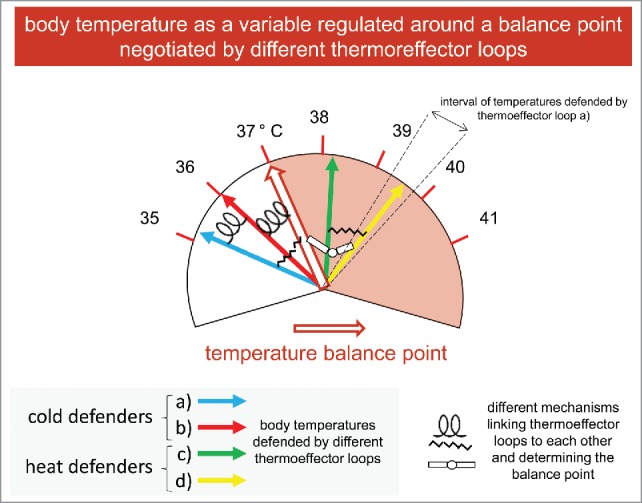
Thermoregulation in a non-unified system.

Since I did not know how to explain these new concepts (not my rough and rude metaphoric approximations to those concepts) to students and residents attending the courses I teach, I started to modify the slide I used in the past, and came out with Figure 2. In the new slide, there is not a temperature set point any more. There is instead a “temperature balance point,” whose position on the scale is determined by the different forces (i.e., the different thermoeffectors loops, depicted as arrows of different colors) pulling on it in different directions, and so negotiating a balance point. As far as the links between the different arrows are concerned, I decided to use different symbols to mean that these links, at multiple levels (from brain circuits to transcription factors) are or may be different. For the sake of clarity, only a few thermoeffector loops and a few links are shown in the figure. Some thermoeffector loops (the blue and red arrows), when activated, would bring down (i. e. lower) the balance point. These would be the cold defenders, that counteract any rise in body temperature. Other thermoeffector loops (the green and yellow arrows), again, when activated, would bring up (i. e. rise) the balance point. These would be the heat defenders, that counteract any decrease in body temperature. Each thermoeffector loop defends the body temperature indicated by the arrow, i. e. it acts against any change from that given temperature. Actually, each thermoeffector loop defends not a single temperature value, but an interval of temperature values. In Figure 2, for the sake of clarity, this is shown only for one thermoeffector loop (d, in yellow).

## The thermoeffector loops

So, body temperature is regulated by the interaction(s) between different thermoeffector loops. But what is a thermoeffector loop? The concept can be obvious to me: a poly-synaptic and multi-layered (at a cranio-caudal level of organization) reflex arch, made of an afferent pathway, which, starting from thermo-receptors and primary afferent sensory neurons, relays the signals (through different relay stations) to thermoeffector neurons located rostrally, in the hypothalamus. Through multiple relay station these thermoeffector neurons would control the activity of peripheral thermoeffectors. Some components of both the afferent and efferent branches of this loop could and would be shared between different loops.

**Figure 3.**
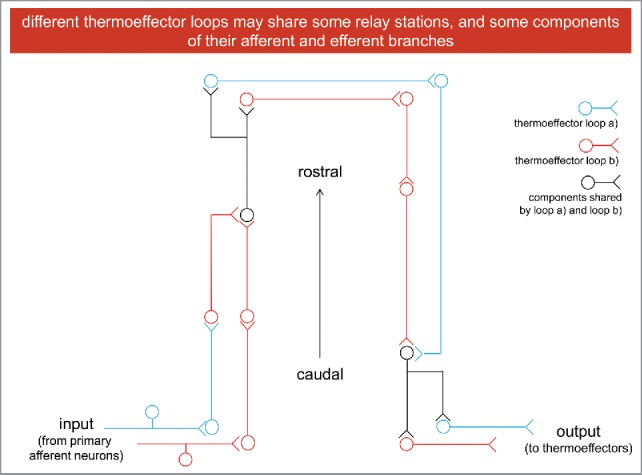
Thermoeffector loops include afferent and efferent branches.

But I am not sure the concept of a thermoeffector loop would be so obvious to the students attending the courses I teach (I always have them in my mind, when preparing a lecture). So, I started from a figure from a paper by Andrej Romanosvky and his collaborators,^5^ but I got rid of all the details (for instance, anatomic nomenclature) to (try to) deliver the only message that reflex, but integrated arches, share some of their multi-components (Fig. 3). In my lecture on thermoregulation, after this general and schematic diagram, I would show another slide, for instance the original figure I was referring to a few lines above. Anatomical and functional details of (at least some) thermoeffector loops would be shown here.

Then there are the “details,” which are actually far from details. And, anyway, are not devil and God always both found in details? For instance:

## Thermoeffector loops have different and specific thresholds of activation

**Figure 4.**
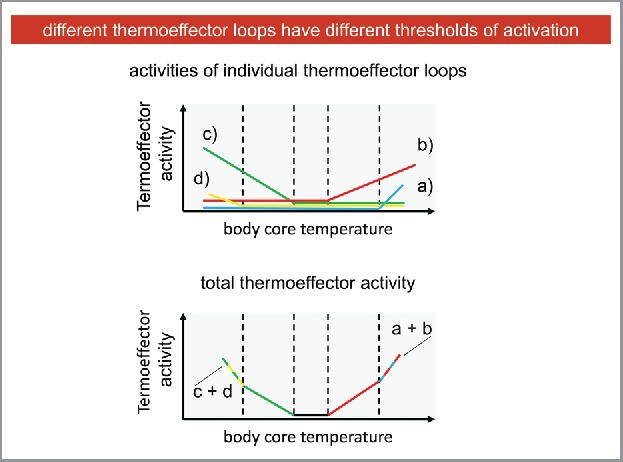
Different thermoeffector loops have different thresholds of activation.

Since, as I explained at the beginning of this paper, I am not a thermophysiologist, I learned from my friends thermophysiologists that different thermoeffector loops are activated at different temperatures. To deliver this concept, I started again from a figure (Fig. 23.3) in a chapter on thermoregulation written a few years ago by Andrej Romanovsky^4^ for a textbook of medical physiology, and I made a few changes to it. The upper part of Figure 4 shows that the 4 thermoeffector loops depicted in Figure 1 (with the same colors and letters) increase their activity at different temperatures, i. e. increase their activity when body temperature is increased above or decreased below a specific (threshold) value. I put the cold defenders (which contrast any increase in body temperature by cooling the body) on the right, to mean that they are activated when body temperature increases: thermoeffector b) would be activated first, for a lower increase, while thermoeffector a) would be activated later, for a larger increase. Symmetrical considerations can be made for the heat defenders c) and d).

The lower part of Figure 4 shows how the 4 thermoeffector loops work together in maintaining body temperature: when body temperature is maintained between the 2 central dashed lines (i. e. it is around the balance point) no thermoeffector loop is activated (i. e. we would be here in the thermoneutral zone, where no temperature increasing, nor decreasing mechanisms are activated). When temperature rises above the first threshold, thermoeffector loop b) reacts against this change, becoming activated. When body temperature goes beyond the second threshold (the dashed line on the far right), thermoeffector loop a) is recruited, and the 2 thermoeffector loops work in cooperation. Again, symmetric considerations can be made for the heat defenders c) and d).

**Figure 5.**
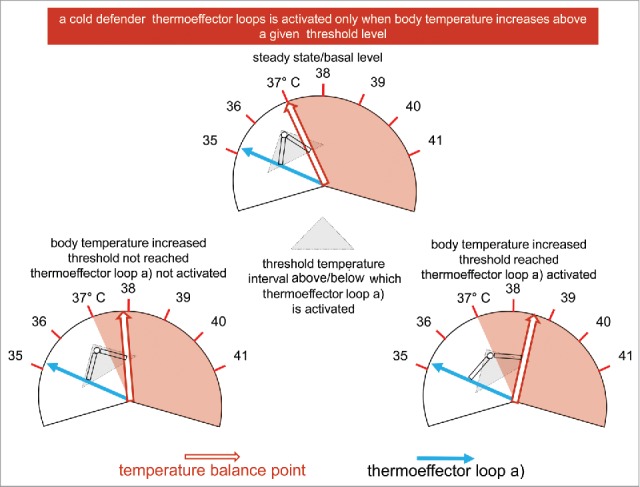
A thermoeffector loop is activated only when a given threshold is passed.

Since I am one of those people who thinks that “a picture is worth a thousand words,” I tried to illustrate the concept that “thermoeffector loops have different thresholds of activation” also in another slide (Fig. 5). For the sake of clarity and simplicity, only one thermoeffector loop (a cold defender) is depicted here. The fundamental idea in this slide is that there is always (by definition) a difference between the body temperature negotiated in any given moment by the different thermoeffector loops (and indicated by the value of the balance point), and the temperature defended by a specific thermoeffector loop: this specific thermoeffector loop will be activated only when the difference between the measured temperature and the defended temperature will pass a threshold. Coming to the slide, starting from a steady-state/basal level in the central upper part of the figure, the bottom left part of the figure illustrates the situation in which the value of the temperature balance point is increased (due to the activation of any mechanism, which we are not interested to discuss here, able to increase body temperature), but the threshold for the activation of thermoeffector loop a) is not reached and this effector is not activated. In the figure at the bottom right, the value of the temperature balance point is increased further, the threshold is passed, and the cold defender thermoeffector loop a) is activated. For any further (by definition supra-threshold) increase in the measured temperature, the difference between the measured temperature and the temperature defended by the thermoeffector loop will increase (and this would be represented as a further stretch of the link between the 2 temperatures), and the activity of the thermoeffector loop contrasting this change will increase accordingly.

The concept that the different thermoeffector loops are activated at different thresholds is very important within the frame of thermoregulation as a non unified system. Thinking of thermoregulation as organized around a set point, hardwired within a hypothalamic control center, it would be the control center, anytime a difference between the measured temperature and the set point is detected, to orchestrate and coordinate the call into action of the different peripheral thermoeffectors. I have (and want) to say that I find very convincing what Andrej Romanovsky writes^4^: there is no need for a central command to account for the recruitment of different thermoeffectors at different, specific and sequential times. Simply, and of course this is pretty far from being “simple,” thermoeffector loops evolved in such a way that they have different thresholds of activation: the effectors which are less energetically costly in defending a temperature (for instance, vasoconstriction) have a lower threshold, and as such are recruited first. The more expensive effectors (for instance, shivering) are recruited later, if the first defense line was not sufficient. Simple is better, or at least this is what I came to think. As Occam taught, having a razor handy (to cut what is not necessary), it is always useful. The sharper the razor, the better.

## When body temperature changes

**Figure 6.**
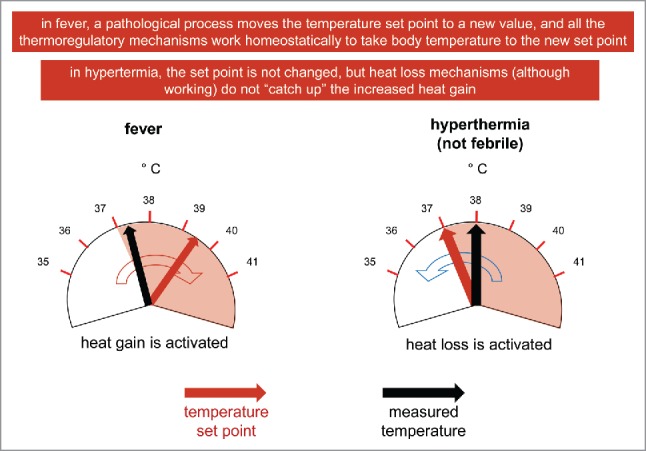
Fever and (not febrile) hyperthermia in a unified model of thermoregulation.

Given that any increase in body temperature is defined as hyperthermia,^4^ I always thought it is important, with the students attending the course I teach, to conceptually make a difference between febrile and non-febrile hyperthermia. To illustrate this point, in the past I used the slide in Figure 6: in fever (left part of the figure), a pathological process moves the temperature set point to a new and higher value, and all the thermoregulatory mechanisms work homeostatically to take body temperature to the new set point. A patient, during the rising phase of fever, would lie in bed, under a blanket (in behavioral thermoregulation), and would shiver. On the contrary, during non-febrile hyperthermia (right part of the figure), the body would accumulate so much heat (either due to increased internal production – for instance during strenuous physical exercise, or to increased ambient temperature) that the heat losing mechanisms, although working perfectly and at full throttle, do not “catch up,” and body temperature is increased. The set point is not altered in this situation, during which a person (for instance a marathon runner) would profusely sweat.

**Figure 7.**
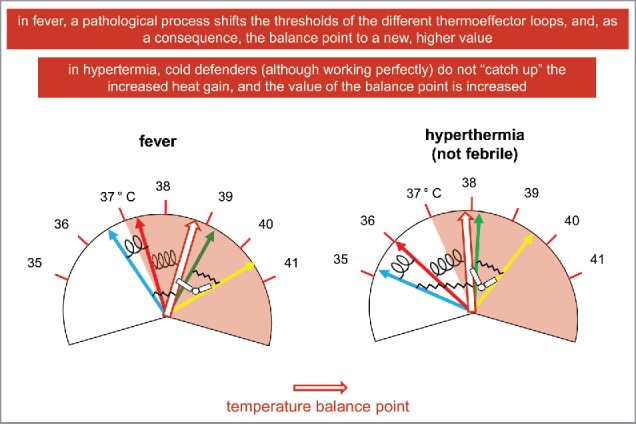
Fever and (not febrile) hyperthermia in a non-unified model of thermoregulation.

But we are hypothesizing not to have a set point and a central commander. I learn^3^ that in fever, a pathological process shifts the thresholds of the different thermoeffector loops, and, as a consequence, the balance point to a new, higher value. I tried to illustrate this situation in the left part of Figure 7. On the other hand, in (non febrile) hyperthermia, there is an increase in ambient temperature and/or in internal heat production, that pull the balance point of body temperature to a higher value (right part of Fig. 7). The difference between the new value of the balance point and the temperature defended by the cold defenders is increased (as shown by the stretched links in this part of the figure). If this difference passes a threshold, these thermoeffector loops are activated, but their activation is not sufficient to counteract the heat gain.

Not for lack of interest, but only for the sake of brevity, I will not discuss hypothermic conditions.

## Teaching thermophysiology to (busy) non-thermophysiologists

I am perfectly aware that, in preparing this lecture of mine on thermoregulatory mechanisms, I very often over-simplified many concepts. I just hope that my over-simplifications did not take me to make any wrong, or misleading statement.

I am perfectly aware of the possible errors in my explanation, but, on the other hand, I also think that nowadays, the amount of information (and hopefully knowledge) medical students, and residents, and, later in time, of course physician (but, personally, my involvement in continuous medical education is minimal) must, if not master, at least handle is vast and impressive. And so we have the responsibility to make as simple as possible everything that can be made, in some measure, simple. Students and residents and physicians cannot get lost in details that are of interest only to the specialists. Which is the practical relevance of the “thermoregulation without a thermostat” model for somebody working in an emergency department and treating for instance a heat stroke? This is the important message to deliver; the details of the theory can be skipped. For all these reasons, teaching in a medical school, I also focus only on conditions occurring in humans, not discussing other conditions occurring in the vast animal kingdom such as, for instance, hibernation.

I know that I have just finished stating that we (probably) need to make the subjects we present in our lecture as simple as possible, but now I would like to (try to) contradict myself, saying that maybe we are digesting the matters we present our students too much. Let me use just one example: scientific illustrations. When I was in medical school (and of course it was the last century), illustrations in my textbooks were mostly little, tiny, black and white thumbs, that often were just graphs reporting original data from primary papers. Now, some illustrations (in textbooks, as well as in prestigious review journals) are almost gorgeous, as far as both digestion of data and their representation are concerned. But making data and concept clear is different from making them (too) simple. Are we not making the effort of learning, which is a struggle, too easy for our students? Are we not depriving them of something?

I use a lot of metaphors and analogies in my teaching. I am convinced that metaphors and analogies may be potent tools in science. But, as I always tell the students attending my courses, metaphors and analogies can also be very (the most?) dangerous tools. Even more when teaching: students have, by definitions, less critical instruments. Metaphors can/may (sometime) shed (some) light, never explain “everything.” For instance, representing in Figure 2 the thermoeffector loops as arrows pointing to a fixed temperature, although a range of values of defended temperatures is also shown, is pretty naïve, and simplistic too. Metaphors should come with a warning: please “handle with (great) care.” On the other hand, an Italian writer I love, Cesare Pavese, once wrote that “words are poor things, but they are all we have.” I could almost say something similar of metaphors.

## From top-down to a bottom-up models

Being a physiologist, of course I am not an epistemologist, but thinking of the model of thermoregulation as a non unified system, made me think of a shift between models in a different field, which I think I know kind of better, the field of sleep controlling brain circuits. In this field, the idea, based on the great, positivistic neurology of the XIX century, that we have a “sleep center” and a “waking center” (like the Broca's and Wernicke's areas), although pursued for a long time, was also abandoned from such a long time that I do not even need to quote a reference here. Later the idea evolved that sleep-wake cycle is regulated by a network (or web) of interacting sleep- and waking-neurons, located in specific brain nuclei, and delivering the resulting command and message to the rest of the brain and then to the body: sleep would be a phenomenon of the whole organism, governed by central control mechanisms.^2^ Over the years (and at this point not so few years), a different hypothesis developed, and here I will directly quote from a review of this model: according to this model, sleep would be a “fundamental property of neuronal networks and is dependent on prior activity in each network. Such local-network sleep might be initiated by metabolically driven changes in the production of sleep-regulatory substances.”^2^ Moreover, “sleep-like states of individual cortical columns can be synchronized through humoral and electrical connections,” so “that whole-organism sleep occurs as an emergent property of local-network interactions.”^2^

Although there are of course differences between the two “new” models of sleep and body temperature regulation, in this move from a model in which a physiologic process (regulating body temperature or sleep) is originated from and controlled by a central command, in a top-down organization, to a model in which the same process is originated from and is controlled by (I could say emerge and result from) the interaction between different forces and processes, in a bottom-up organization, I see different analogies.

Maybe we do not need any top organizer (i. e., by definition, a supra-structure), when things “down there,” in a process that took a few millions years, got organized by themselves so nicely. Is not it that maybe we feel the need of somebody above because we are scared to find out that, like Albert Camus' Sisyphus, we are, down here on this earth, simply and plainly alone? But here I crossed a line that I should not have crossed, so I will stop here.

I will only add that I am pretty sure that a similar shift between top-down to bottom-up models already occurred or is occurring in other scientific fields, of course also outside the field of biomedicine and even life sciences, and if colleagues, more knowledgeable than I, will be willing to contribute to this discussion, I will be very grateful.

## Post scriptum

Alessandro Manzoni thought to have 25 readers for his “The Betrothed.” Maybe some of the handful of readers of this paper noticed that I never used any abbreviations and/or acronyms throughout the manuscript. I hate acronyms. We all use too many acronyms and abbreviations. Nowadays (most) acronyms are unbelievably long and very complicated, to the point to be, in my opinion, (completely) meaningless (if not, in some cases, even potentially misleading or deceptive). I do not want to offend anybody (the other way around), but, since this is a journal devoted to (body) temperature regulation, and thermal and pain sensitivities are related, I will use this example, just by chance: TREK stands for “TWIK-related K^+^ channel,” but TWIK, in turn, stands for “tandem pore domain weak inwardly rectifying K^+^ channel” (where I get lost). At the end, TREK would read “tandem pore domain weak inwardly rectifying K^+^ channel-related K^+^ channel.” I could be very easily and completely wrong, but could not a completely unrelated “tag/name” of any sort have worked as well (or better)? I wonder (with some scare) how the nicotinic receptor would be called if discovered today. I am worried that (at least part of) the point is that nowadays a molecular biologist works on the structure of a protein, having no way to determine its function. But this colleague has anyway to give a name to this “thing,” and probably will use an abbreviation based of some descriptor of its structure. At the same moment, somewhere, an electrophysiologist will be studying the properties of a membrane channel, having no way to know its composition and structure. This other colleague, the electrophysiologist, will also have to find a name for this new (and beloved) creature, and probably will use an abbreviation based on some descriptor of its electrophysiological properties. At a certain point, the 2 colleagues and the 2 different forms of knowledge will meet. Having different expertise, and using different languages and words, how long will it take them to find out that they are speaking of the same thing? And, at that point, a new abbreviation to name the new “thing” will be born. It is possible it will be very long.

In the lectures I give, I try to skip acronyms and abbreviations as much as possible, since, in my personal experience, after the fifth acronym in a lecture, most of the students are lost, too busy trying to remember what is behind that acronym.
